# The SARS-CoV-2 Nucleocapsid Protein and Its Role in Viral Structure, Biological Functions, and a Potential Target for Drug or Vaccine Mitigation

**DOI:** 10.3390/v13061115

**Published:** 2021-06-10

**Authors:** Zhihua Bai, Ying Cao, Wenjun Liu, Jing Li

**Affiliations:** 1 CAS Key Laboratory of Pathogenic Microbiology and Immunology, Institute of Microbiology, Chinese Academy of Sciences, Beijing 100101, China; baizhihua19@mails.ucas.ac.cn (Z.B.); caoyingor@163.com (Y.C.); liuwj@im.ac.cn (W.L.); 2 Savaid Medical School, University of the Chinese Academy of Sciences, Beijing 100049, China; 3 CAS Key Laboratory of Special Pathogens and Biosafety, Wuhan Institute of Virology, Chinese Academy of Sciences, Wuhan 430071, China; 4 Center for Biosafety Mega-Science, Institute of Microbiology, Chinese Academy of Sciences, Beijing 100101, China

**Keywords:** SARS-CoV-2, nucleocapsid protein, structure, biological function, vaccine

## Abstract

The impact of severe acute respiratory syndrome coronavirus 2 (SARS-CoV-2) on the world is still expanding. Thus, there is an urgent need to better understand this novel virus and find a way to control its spread. Like other coronaviruses, the nucleocapsid (N) protein is one of the most crucial structural components of SARS-CoV-2. This protein shares 90% homology with the severe acute respiratory syndrome coronavirus N protein, implying functional significance. Based on the evolutionary conservation of the N protein in coronavirus, we reviewed the currently available knowledge regarding the SARS-CoV-2 N protein in terms of structure, biological functions, and clinical application as a drug target or vaccine candidate.

## 1. Introduction

Coronavirus (CoVs) can cause a variety of diseases in humans and animals, such as infectious gastroenteritis in livestock, infectious bronchitis in chickens, and the common cold with some mild respiratory symptoms in humans [1,2]. However, in the past two decades, novel deadly CoVs have emerged, causing three infectious disease pandemics in human society, resulting in enormous health threats and disrupting the global economy [3]. Severe acute respiratory syndrome coronavirus 2 (SARS-CoV-2) was first reported in December 2019 and has since spread worldwide, making it the seventh known CoVs to infect humans [4]. The disease caused by SARS-CoV-2 infection is called COVID-19, and is characterized by symptoms, such as shortness of breath or dyspnea, fever, muscle pain, even death in severe cases [5]. The cumulative number of confirmed COVID-19 cases already exceeded 200 million worldwide.

Molecular evolution analysis based on nucleic acid sequence alignment shows that SARS-CoV-2 is a member of the genus *β-Coronavirus* and the subgenus *Sarbecovirus*. The viral genome consists of a positive-sense, single-stranded RNA, which comprises 14 open reading frames (ORFs), encoding 16 nonstructural proteins that make up the replicase complex, nine accessory proteins (ORF), and four structural proteins: spike (S), envelope (E), membrane (M), and nucleocapsid (N) [4,6,7]. Among them, the N protein is highly conserved in the CoVs genus and is one of the most abundant structural proteins in virus-infected cells [8]. The fundamental function of the N protein is to package the viral genome RNA into a long helical ribonucleocapsid (RNP) complex and to participate in the assembly of the virion through its interactions with the viral genome and membrane protein M [9]. In addition, the N protein of the CoVs has been shown to be involved in the host cellular machinery such as interferon inhibition, RNA interference, and apoptosis, serving a regulatory role in viral life cycles [10,11]. Moreover, the N protein is also an immunodominant antigen in host immune responses that can be used as a diagnostic antigen and immunogen [12]. As summarized in this article, numerous studies on the N protein of SARS-CoV-2 to investigate the role of the N protein in viral assembly, replication, and the host immune response regulation, to provide a reference for developing specific immune-based drug and vaccine.

## 2. The Biological Function of the SARS-CoV-2 N Protein

### 2.1. Composition and Structure of SARS-CoV-2 N Protein

**Figure 1:**
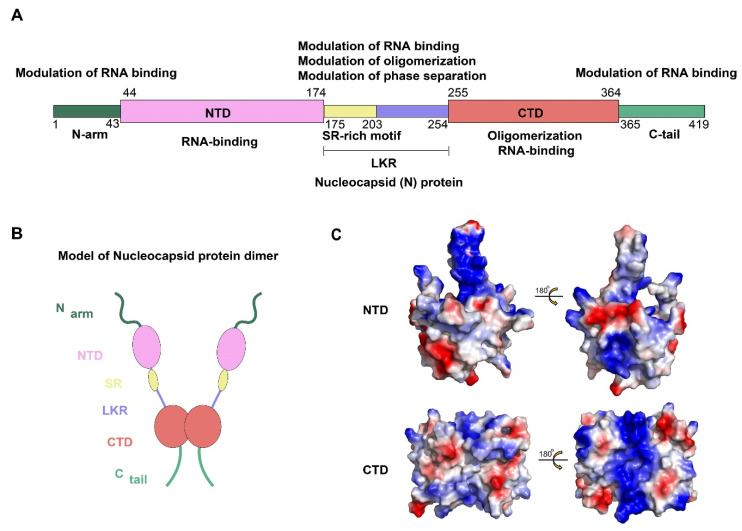
Structural overview of the SARS-CoV-2 N protein. (**A**,**B**) Schematic of the SARS-CoV-2 N protein modular organization. The three intrinsically disordered regions, including the N-arm, central Ser/Arg(SR)-rich flexible linker region (LKR) and C-tail, and the N-terminal domain (NTD) and C-terminal domain (CTD) are illustrated. (**C**) Electrostatic surface of the SARS-CoV-2 N-NTD (PDB ID 6YI3) and N-CTD (PDB ID 7CE0). Blue denotes positive charge potential, while red indicates negative charge potential. All structural figures were prepared using PyMOL.

The N protein of SARS-CoV-2 is encoded by the ninth ORF of the virus and is composed of 419 amino acids. Like other CoVs, the SARS-CoV-2 N protein has a modular organization which can be divided into intrinsically disordered regions (IDRs) and conserved structural regions according to the sequence characteristics [13]. The IDRs include three modules: N-arm, central Ser/Arg-rich flexible linker region (LKR), and C-tail, while the conserved structural regions including two modules: N-terminal domain (NTD) and C-terminal domain (CTD). In the primary structure, NTD and CTD are connected by LKR and are usually flanked by N-arm and C-tail (Figure 1A,B).

To date, the NTD and CTD of the SARS-CoV-2 N protein have been solved and show strongly resembles other CoVs N protein structures [14,15]. For SARS-CoV-2 N protein, each NTD molecule presents a right-handed fist shape. The core subdomain consists of a five-stranded U-shaped antiparallel β-sheet with the topology β4–β2–β3–β1–β5, sandwiched between two short α-helices (α1 before β2 strand and α2 after β5). There is a large protruding β-hairpin ((β2′–β3′) between β2 and β3 as a bridge to connect them, which stands out of the core (PDB ID: 6YI3). In terms of the CTD, it exists as a tight homodimer and displays an overall rectangular slab shape, in which each protomer is comprised of five α-helices, two β-strands, and two 3_10_ helices. The β-hairpin from one protomer is inserted into the cavity of the other protomer, resulting in the formation of the four-stranded, antiparallel β-sheet at the dimer interface. The β sheet forms one face of the slab dimer, while on the opposite face of the dimer, the surface is formed by α-helices and loops (PDB ID: 7CE0). Extensive hydrogen bond interactions between the two hairpins and hydrophobic interactions between the β-sheet and the α-helices make the dimeric structure highly stable [14]. However, due to various reasons, such as difficulty in maintaining protein stability and the highly disordered sequence of IDRs, there are no structures available for any of the full-length N proteins from CoVs [16,17]. Some bioinformatics methods may provide some hints. A recent study compared the IDRs of N protein, as well as other CoVs proteins, between SARS-CoV-2, SARS-CoV, and bat SARS-like CoV, which provide important grounds for a better understanding of the biological functions and structure [18]. Meanwhile, the information regarding the N-IDRs by using a combination of 2D spectra and nuclear magnetic resonance (NMR) is worthy of consideration [19].

### 2.2. Genome Encapsidation: The Primary Function of a Viral N Protein

The fundamental function of the SARS-CoV-2 N protein is to package the viral genome into an RNP particle. Thus, the N protein should have the ability to recognize and bind RNA, which numerous studies have proven [17]. Among the SARS-CoV-2 N-NTD domain, the protruding β-hairpin (β2′–β3′) is composed mostly of basic amino acid residues. Further analysis of the surface electrostatic potential revealed a positively charged pocket at the junction between the basic hairpin and the core structure which served as a putative RNA binding site (Figure 1C) [15]. By building atomic models of the protein: RNA complex, Dinesh et al. proved that both dsRNA and ssRNA bind in a similar manner to the positively charged canyon located between the basic β-hairpin and the core of the N-NTD where the arginine residues (R92, R107, and R149) that directly bind the RNA are located. Previous studies have proven the presence of another RNA binding domain with a positively charged groove in the helix face of the N-CTD dimer [17,20,21]. For the SARS-CoV-2 N-CTD, the positively charged groove consists of K256, K257, K261, and R262 residues (Figure 1C) [14]. In addition, it was reported that both the N-terminal IDR and the LKR have RNA binding activity [22]. While increasing binding affinity, they also enhance the binding allostery, enabling the N protein to bind RNA with high cooperativity [23]. Taken together, this information indicates that the NTD, the CTD, and some disordered regions of N protein can bind RNA cooperatively to promote RNP packaging.

Another crucial property of the N protein for genome encapsidation is its ability to self-associate. The N-CTD of other β-CoVs was confirmed to self-associate to form an oligomer (dimer, trimer, tetramer, or hexamer, in a concentration-dependent manner) [16,24,25,26]. Similarly, the high-resolution crystal structure of SARS-CoV-2 N-CTD shows a compact, strand-swapped dimer in solution [14]. Most important, it was found that the C-terminal domain (residues 365–419) can also self-assemble and further mediate N protein tetramer formation [27]. Studies have also shown that the Ser/Arg-rich portion of the LKR domain is highly phosphorylated, which not only affects the nucleocytoplasmic shuttle of the N protein but also regulates the self-association of the N protein [28]. Further experiments have proven that the phosphorylation of LKR reduces the total positive charge of N protein and thereby regulates the oligomerization of the N protein through the electrostatic effect [29]. Therefore, the assembly of full-length N protein into helical filaments is a complex biological process, which is mediated by cooperative interactions among several interfaces.

### 2.3. N Protein Undergoes Liquid-Liquid Phase Separation to Enhance Viral Transcription and Assembly

Liquid-liquid phase separation (LLPS) is a common cellular process to organize biological material into compartments. During the process of virion assembly, it is necessary to format dense protein-nucleic acid compartments that sequester host cell proteins as a means of protection from the host immune system and to locally concentrate viral components to increase the efficiency of replication [30]. Given that it contains both IDRs and RNA-binding domains, the N protein displays the hallmarks of proteins that undergo LLPS [31]. To date, several groups have proved that the N protein of SARS-CoV-2 can phase separately with various RNAs in vitro and its phase behavior is tuned by pH, salt, and RNA concentration [32,33,34,35,36,37]. Meanwhile, it has been revealed that the SR-phosphorylation of N proteins modulate RNA-induced phase separation. The unmodified N protein forms partially ordered gel-like condensates based on multivalent RNA-protein and protein–protein interactions, which facilitates assembly of the nucleocapsid. While the phosphorylated SR region reduces these interactions, generating a more liquid-like compartment for viral genome processing [33]. In addition, a recent observation suggests that viral RNA sequence and structure regulate N protein condensation, depending on their binding patterns. LLPS-promoting sequences are located at 5′ and 3′ ends of the genome, suggestive of a genome packaging role, while other genomic regions promote condensate dissolution, potentially preventing aggregation of the large genome [34].

However, although it was clear that the N protein would undergo LLPS and some regulatory elements have been found, how phase separation might relate to a single-genome packaging, although RNA compaction has not been clearly described in detail. So far, some models have been proposed. One model is similar to that proposed in the measles virus studies [38], in which the process of phase separation is decoupled from genome packaging. In this model, the SARS-CoV-2 N protein and genome RNA (gRNA) phase separate in the cytosol. Subsequently, a discrete pre-capsid state forms within condensates, while gRNA condensation occurs through association with a helical nucleocapsid. Upon maturation, the pre-capsid is released from the condensate and undergoes subsequent virion assembly by interacting with the membrane-bound structural proteins (M, E, and S) at the ER-to-Golgi intermediate compartment (ERGIC). Another attractive model interpretation is that the N protein has evolved to drive genome compaction for packaging. In this model, a single-genome condensate forms through dynamic multivalent interactions between the N protein and gRNA. During this process, condensate-associated N proteins exchange with soluble N proteins, so the interactions that drive compaction are heterogeneous and dynamic. Subsequently, the single-genome condensate undergoes maturation, leading to virion assembly. Further, the mature condensate then interacts with M protein directly [39]. Both models require more experimental data to support their reliability. Of note, it has been demonstrated that LLPS of SARS-CoV-2 N protein promotes cooperative association of the RNA-dependent RNA polymerase complex with polyU RNA in vitro, which suggests that SARS-CoV-2 uses LLPS-based mechanisms similar to transcription hubs in cellular nuclei to enable high initiation and elongation rates during viral transcription [32,40].

### 2.4. Regulation of the Host’ Immune Response

The innate immune system functions as the first line of host defense against microbial infections by recognizing and removing infected cells while coordinating adaptive immune responses during viral infection [41]. To replicate effectively in host cells, viruses have evolved a variety of antagonistic strategies to circumvent innate immune detection, such as glycosylation to shield essential invasive sites, reduction of the cytosine phosphate guanosine (CpG) levels in the genome, and generation of viral proteins to interfere with anti-viral responses [42]. Together these strategies allow widespread and effective infection. Current studies showed that the N protein also plays a part in this game.

**Figure 2:**
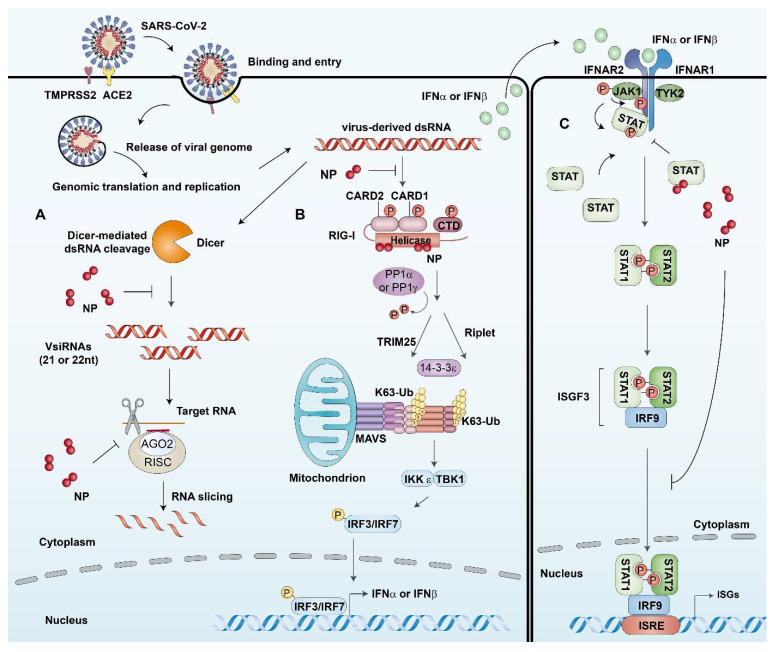
The host cell’s innate immune processes involving the N protein. (**A**) N proteins act as viral suppressors of RNA interference (RNAi) in host cells. In the initiation step, the dsRNA in infected cells could be sequestrated by N proteins, which prevent the recognition and cleavage of viral dsRNA by Dicer. In addition, the N protein can suppress siRNA-induced RNA degradation in the effector step. (**B**) N proteins interact with RIG-I and repress RIG-mediated IFN-β production. N proteins can interact with RIG-I through the helicase domain of DExD/H-box helicases, which has ATPase activity and plays an important role in the binding of immunostimulatory RNAs. Therefore, SARS-CoV-2 N proteins suppress the IFN-β response by targeting the initial step of IFN activation. (**C**) N proteins antagonize type I IFN signaling by suppressing phosphorylation and nuclear translocation of signal transducer and activator of transcription 1 and 2 (STAT1 and STAT2). The binding of secreted type I IFN to their receptors on neighboring cells can trigger phosphorylation of pre-associated Janus kinase 1 (JAK1) and tyrosine kinase 2 (TYK2), which in turn phosphorylates the receptors. This leads to the recruitment and phosphorylation of STAT1 and 2. In SARS-CoV-2 infected cells, N proteins can interfere with the interactions of STAT1 with JAK1 and STAT2 with TYK2 by competitively binding to STAT1/STAT2 and in turn inhibit their phosphorylation. Therefore, N proteins further reduce the subsequent nuclear translocation of the IFN-stimulated gene factor 3 (ISGF3) transcription complex and inhibiting the expression of IFN stimulated genes (ISGs).

In host cells, there is a cell-intrinsic antiviral immune defense mechanism, termed RNAi, which can lead to the destruction of the viral genome to inhibit viral replication [43]. As a countermeasure, viruses encode viral suppressors of RNAi (VSRs) to antagonize the RNAi pathway. In past studies, the SARS-CoV N protein has exhibited a VSR activity in mammalian cells [44]. Intriguingly, cells infected with SARS CoV-2 also exhibited the same phenomenon and SARS CoV-2 N protein can antagonize RNAi in multiple steps. In the initiation step, the dsRNA in infected cells could be sequestrated by the N protein, which probably prevents the recognition and cleavage of viral dsRNA by Dicer. In addition, the N protein can suppress siRNA-induced RNAi in cells, implying the antagonistic effect in the effector step [45]. This suggests that using N protein as the VSR is a common immune evasion strategy for coronaviruses (Figure 2A). It is well known that infection by various RNA viruses activates the RIG-I-like receptor pathway and initiates the expression of IFN-β, which is one of the main mechanisms of the host innate immune defense [46,47]. It has been identified that SARS-CoV-2 ORF6, ORF8, and N are potent interferon antagonists [48,49]. Further research found that the N protein could inhibit IFN production to varying degrees in different ways. It worth noting that N protein interacted with RIG-I through the Helicase domain, which plays an important role in the binding of immunostimulatory RNAs, suggesting that SARS-CoV-2 N protein could suppress the IFN-β response through targeting the cellular RIG-I and RNA recognition, the initial step of IFN activation (Figure 2B) [50]. The other part of the IFN-mediated antiviral pathway is the Janus kinase (JAK)-signal transducer and activator of transcription (STAT) pathway, which activates the expression of a set of interferon-stimulated genes (ISGs) to establish the host antiviral state. A recent study found that the SARS-CoV-2 N protein also exhibited a significant inhibitory effect on this process. Specifically, the N protein interfered with the interactions of STAT1 with JAK1 and STAT2 with TYK2 by competitively binding to STAT1/STAT2 and, in turn, inhibiting their phosphorylation and subsequent nuclear translocation in the 293T cells. Furthermore, in the HepG2 cells expressing N protein and infected with SARS-CoV-2, it was found that the phosphorylation of STAT1 and the mRNA levels of ISG15 were reduced and the viral RNA replication was significantly elevated. Taken together, these findings indicated that the N protein could efficiently enhance the replication of SARS-CoV-2 by suppressing the phosphorylation and nuclear translocation of STAT1/STAT2, subsequently inhibiting the expression of ISGs (Figure 2C) [51]. In summary, emerging evidence about the SARS-CoV-2 N protein, and comparison with SARS-CoV reveal the many strategies used to evade the innate immune response. These facilitate widespread viral replication and probably lead to exacerbation and hyperinflammation of the innate immune response once triggered.

## 3. Clinical Applications of the SARS CoV-2 N Protein

### 3.1. N-Protein as a Diagnostic Marker

Prompt detection is essential to limit the spread of pathogens. The availability of the complete genomic sequence of SARS CoV-2 has facilitated the development of a variety of diagnostic tests for SARS-CoV-2. Reverse transcription polymerase chain reaction (RT-PCR) has been used as a rapid diagnostic test during the epidemic [52]. However, the sensitivity of viral RNA testing varies depending on the timing of testing relative to exposure, which could lead to false-negative results [53]. Thus, more and more laboratories pay attention to serological tests.

Among the four CoVs structural proteins, the S and N proteins are the main immunogens [54]. There have been several serological tests showing that S and N induced a strong antibody response in hosts [55,56]. During the detection process, it was found that the detection rate of N protein was higher than that of S protein in PCR-positive patients [57]. Hence, it is a feasible method to use N proteins for serological tests or combined N and S proteins as capture antigens to increase the sensitivity of this assay. However, one question raised is that the test for specific antibodies against SARS-COV-2 in the serum will appear positive only about 7 days after infection or later, making it difficult to detect the infection at an early stage. Given this situation, it is necessary to explore the diagnostic value of SARS-CoV-2 proteins in the early stages of SARS-COV-2 infection. Several studies have detected serum N protein level in SARS-COV-2 infected patients and analyzed the correlation with serum N protein antibody level using the commercial kit. Based on the CUT-OFF value determined from the receiver operating characteristic (ROC) curve, the specificity of the SARS-COV-2 serum N protein detection was 96.84%, and the sensitivity was 92% before the appearance of antibodies, suggesting that the detection of SARS-COV-2 serum N protein has a high diagnostic value for infected patients before the appearance of antibodies, and shortens the window of serological diagnosis [58]. Meanwhile, several laboratories have tried to identify the immunodominant epitopes of N protein and develop specific monoclonal antibodies that can be used in ELISA. Amrun et al. identified four immunodominant epitopes: S14P5, S20P2, S21P2, and N4P5, on the S and N viral proteins. IgG responses to all identified epitopes displayed a strong detection profile, with N4P5 achieving the highest level of specificity (100%) and sensitivity (>96%) against SARS-CoV-2, suggesting the feasibility of developing mAbs to these epitopes alone or in combination used in ELISA to detect SARS-CoV-2 [59].

Taken together, all these data support the notion that the N-protein could be used as an efficient diagnostic tool for detection of SARS-CoV-2 infection and the specific detection methods of N protein should further be validated in more patient samples.

### 3.2. N Protein: As a Therapeutic Target

**Figure 3:**
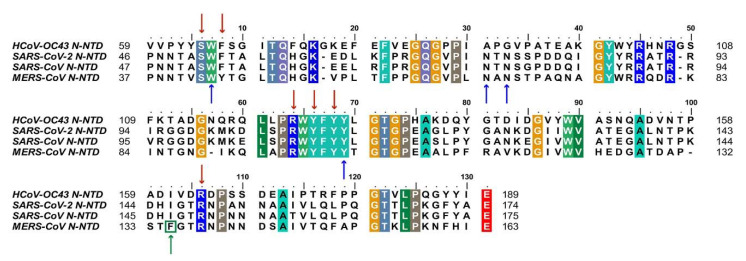
Sequences alignment of four CoVs N-NTD. Multiple sequence alignment of HCoV-OC43 (UniProtKB: P33469), SARS-CoV-2 (UniProtKB: P0DTC9), SARS-CoV (UniProtKB: P59595), MERS-CoV (UniProtKB: K9N4V7). The highly conserved residues were filled with colors. Red arrows indicate conserved RNA binding sites. Blue arrows and green arrow indicate conserved and mutant residues for the non-native interaction interface, respectively. HCoV-OC43, human coronavirus OC43; SARS-CoV-2, severe acute respiratory syndrome coronavirus 2; SARS-CoV, severe acute respiratory syndrome coronavirus; MERS-CoV, Middle East respiratory syndrome coronavirus. (The fill color selected in this figure legend is the default setting of the BioEdit software.).

Despite extensive research on COVID-19, there is currently no effective treatment available for clinical use. Based on the conservation of CoVs N protein in evolution and its key role in viral replication, it is a promising target for drug discovery. Firstly, since the RNA binding activity of N protein is pivotal to viral RNP formation and genome replication, blocking the RNA binding of N-NTD has been proven to be a considering strategy. To date, there have been some small compounds targeting other CoVs considered as candidate inhibitors for SARS-CoV-2 by virtual screening. For example, the compounds PJ34 and H3, which targeted the RNA binding site of N-NTD, can inhibit HCoV-OC43 replication [14]. Notably, the key residues that are involved in the RNA binding interactions, including S51, F53, R107, Y109, Y111, and R149 (in SARS-CoV-2 N-NTD numbering), are conserved, suggesting potential development possibility (Figure 3) [60,61].

In addition, blocking normal N protein oligomerization or triggering abnormal RNP formation is also an attractive inhibitory strategy. More recently, Lin et al. identified 5-benzyloxygramine (P3) is a novel inhibitor for MERS-CoV by virtual screening. This compound could mediate MERS-CoV N-NTD non-native dimerization and induce N protein aggregation. The structure-based study showed that P3 targets the non-native interface of N-NTD dimers and simultaneously interacts with the hydrophobic pockets in both N-NTD protomers. It was demonstrated that P3 was able to replace the vector-fusion residues of promoter 2 to occupy its binding cavity in promoter 1 under the legend free condition, which, in turn, stabilized the dimeric status by triggering massive hydrophobic interactions [14,62]. By comparing the binding sites of P3 in the hydrophobic cavity, it was found that almost all of the residues of the N-NTD involved in the interactions are conserved, except F135 in MERS-CoV, which is replaced by I146 in SARS-CoV-2(Figure 3). Although both residues are nonpolar amino acids, the effect on SARS-CoV-2 replication needs to be further verified. For other viruses, such as the human immunodeficiency virus and influenza virus, the researchers proposed a strategy to inhibit viral N protein oligomerization by developing competing peptides [63,64]. For CoVs, it has been shown that the excessive peptide based on the C-terminal tail sequence can interfere with CTD oligomerization of HCoV-229E N protein and decrease the viral titer, providing a reference for relevant studies on SARS COV-2 N protein [65]. Notably, the LLPS of N protein induced by viral genomic RNA is also a potential target [35]. Slowing viral infection by increasing or decreasing the N protein LLPS is a strategy that could be considered. 1,6-hexanediol, lipoic acid, and aminoglycoside kanamycin, each of which potentially alters LLPS by a representative and distinct mechanism. In terms of SARS-CoV-2, further experiments showed that the formation or the size of condensates could be reduced after treatment with these small molecules [34]. Meanwhile, high-throughput virtual screening is underway, several potential drug candidates have been proposed, and the next focus is on rigorous experimental validation, such as (−)-catechin gallate and (−)-gallocatechin gallate [66] (Table 1).

## 4. Perspectives

Considerable insights regarding the structure and function of the β-CoVs N protein were revealed in the last decades due to the emergence of SARS-CoV and MERS-CoV. Remarkably, the SARS-CoV-2 N protein shares a common modular structure organization including the NTD, CTD, and IDRs with other CoVs. Existing studies have shown that the SARS-CoV-2 N protein is a multifunctional RNA-binding protein, which is not only responsible for packaging viral genomes but also regulates the innate immune response caused by viral infection. However, the quest for understanding how the SARS-CoV-2 N protein, as well as CoVs N proteins in general, forms the RNP and carries out its roles during the viral life cycle is still far from over. Hence, more effort will be required to clarify its role. In addition, compared with other structure proteins, the conserved nature of the N protein in the evolutionary process cannot be ignored, and it is therefore considered an important diagnostic marker and drug target. Recently, inhibition strategies targeting the SARS-CoV-2 N protein have been proposed, such as blocking the binding between the N protein and viral genome or inducing abnormal oligomerization of the N protein to inhibit the correct assembly of RNP, thus affecting progeny viral replication. Meanwhile, experimental validation of drug candidates from high-throughput virtual screening is also progressing.

Unfortunately, the epidemic of SARS-CoV-2 is still ongoing and is likely to become a common pathogen, which makes combating this disease a constant challenge [70]. Therefore, in the long-term, a vaccine to prevent infection is crucial. To date, several COVID-19 vaccines have been approved for the market, all of which were selected from the S protein or the receptor-binding domain (RBD) of S protein as the leading immunogen, based on the critical role of the S protein in the process of virus invasion [71,72]. However, some problems cannot be ignored; primarily the nonsynonymous mutations, which have developed in the S protein as the SARS-CoV-2 epidemic progressed [73,74], which may jeopardize previous vaccine countermeasures. Given this concern, Chen et al. obtained or generated a panel of authentic infectious SARS-CoV-2 strains with sequence variations in the spike gene, including a B.1.1.7 isolate, chimeric Washington strains with a South African (Wash SA-B.1.351), or Brazilian (Wash BR-B.1.1.248) spike gene and isogenic recombinant variants, with designed mutations or deletions at positions 69–70, 417, 484, 501, 614 and/or 681 of the S protein, subsequently using monoclonal antibodies (mAbs), animal immune sera, human convalescent sera, and human sera from recipients of the BNT162b2 mRNA vaccine to evaluate the effects of SARS-CoV-2 strain variation on antibody neutralization. Many highly neutralizing mAbs, most convalescent sera, and mRNA vaccine-induced immune sera reduced the inhibitory activity against viruses containing an E484K spike mutation [75]. Hence, adjusting the gene sequence of the spike antigen to prevent the vaccine from losing protective ability in vivo may need to be considered.

Meanwhile, more attention should be paid to the more conservative N protein. The N protein of CoVs was highly immunogenic and is expressed abundantly during infection [76]. High levels of IgG antibodies against the N protein have been detected in sera from SARS-CoV and SARS-CoV-2 patients [55,56,77], and the N protein is a representative antigen for the T-cell response in a vaccine setting, inducing SARS-CoV and SARS-CoV-2-specific T cell proliferation and cytotoxic activity [78,79,80]. Immune monitoring of patients recovering from SARS-CoV infection showed SARS-CoV-specific antibodies dropped below the limit of detection within 2 to 3 years [81], whereas the specific memory T cells have been detected even 11 years later [82]. Thus, more importantly, the induction of long-term T cell immune memory must be considered in vaccine design. It is exciting to discover that SARS-CoV recovered patients possess long-lasting memory T cells that are reactive to the N protein of SARS-CoV 17 years after the outbreak of SARS in 2003, and these T cells displayed robust cross-reactivity to the N protein of SARS-CoV-2 [83]. Given the prior exposure to other human coronaviruses may confer some protection, Oliveira et al. predicted major T cell epitopes of the SARS-CoV-2 N protein and compared with the other CoVs that infect humans to find conserved motifs [84]. Notably, it has been found that a potential T cell cross-reactivity region within the SARS-CoV-2 N protein position 102–110 amino acids that traverse multiple human CoVs exists. Vaccination strategies designed to target these conserved peptides may elicit cross-reacting T cell responses among different CoVs. In addition, a DNA-based vaccine platform examined the safety and immunogenicity of the SARS-CoV-2 N gene as an antigen and found that the N protein plasmid could induce anti-N titers of 104 to 105 after a boost in New Zealand White rabbits. Further, when C57BL/6 mice were immunized with N protein in adjuvant or 50 μg of DNA, specific T cell responses could also be detected [85]. These reports offer important and timely insights relevant to the SARS-CoV-2 N protein as a vaccine target. Compared with other potential SARS-CoV-2 antigens, the N antigen has some distinct advantages, including the conservation of the N protein sequence, the expanding knowledge of its genetics and biochemistry, and its strong immunogenicity. Hence, considering the N antigen as a vaccine candidate for SARS-CoV-2 and/or including N antigen in spike-related vaccine design, adds the benefits of increasing immunogenicity and ensures a more future-proof vaccine strategy. Regardless of the considerable areas remaining to be explored about the N protein of SARS-CoV-2, more work is needed to improve our understanding of the biology of SARS-CoV-2 and may contribute to the design of better prevention tools.

## Figures and Tables

**Table 1:** β-CoV inhibitors target N protein.

Compounds	Target Domain or Process	Mechanism	Reference
PJ34, *N*-(6-oxo-5,6-dihydrophenanthridin-2-yl) (*N*,*N*-dimethylamino) acetamide hydrochloride	NTD	Reduce RNA binding	[14,67]
H3, 6-chloro-7-(2-morpholin-4-ylethylamino) quinoxaline-5,8-dione	NTD	Reduce RNA binding	[61]
(−)-catechin gallate	NTD	Reduce RNA binding	[66]
(−)-gallocatechin gallate	NTD	Reduce RNA binding	[66]
P3, 5-benzyloxygr- amine	CTD	Induce abnormal dimerization	[62]
1,6-hexanediol	LLPS	prevent condensate formation	[68]
Lipoic acid	LLPS	Reduce smaller condensate	[68,69]
